# Phenotypic spectrum of *FGF14*-related late-onset ataxia: predominant tremor and cognitive decline as key features of SCA27A

**DOI:** 10.1007/s00415-026-13813-1

**Published:** 2026-05-13

**Authors:** Meret Möller, André Fienemann, Joanne Trinh, Christoph Much, Björn H. Falkenburger, Iñaki Schniewind, Yorck Hellenbroich, Norbert Brüggemann, Christine Klein, Alexander Balck

**Affiliations:** 1 Section for Movement Disorders, Department of Neurology, University of Lübeck, University Hospital Schleswig-Holstein, Campus Lübeck, Lübeck, Germany; 2 Institute of Neurogenetics, University of Lübeck, Lübeck, Germany; 3 Department of Neurology, University Hospital Carl Gustav Carus, TUD Dresden University of Technology, Dresden, Germany; 4 German Center for Neurodegenerative Diseases (DZNE), Dresden, Germany; 5 Department of Human Genetics, University Hospital Schleswig-Holstein, Lübeck, Germany

Dear Sirs,

Although panel diagnostics and whole-exome sequencing are standard practices, both can overlook relevant spinocerebellar ataxia (SCA) types, particularly those caused by intronic repeat expansions. Due to these limitations and the broad phenotypic and genotypic spectrum of autosomal-dominant spinocerebellar ataxias [1], whole-genome sequencing is the preferred diagnostic tool for this condition.

Pathogenic variants in the fibroblastgrowthfactor14 (*FGF14*) gene, located on chromosome 13q33.1, cause spinocerebellar ataxia type 27 (SCA27) with a heterogeneous spectrum of phenotypic features, classified into two subtypes: the ultrarare SCA27A alongside the recently identified and the more common SCA27B. Both forms are inherited in an autosomal-dominant fashion. *FGF14* is involved in regulating brain sodium channels, particularly in the cerebellum, thereby modulating the spontaneous and evoked firing of Purkinje cells [2]. SCA27A is typically associated with loss-of-function pathogenic variants or small structural variants in *FGF14*, leading to haploinsufficiency [3, 4]. Some patients have deletions encompassing *FGF14* and integrin, beta-like 1 (*ITGBL1*) [5–7]. In contrast, SCA27B is caused by intronic GAA trinucleotide repeat expansions within intron 1 of *FGF14*.

The core phenotype of SCA27B comprises slowly progressive late-onset cerebellar ataxia and eye movement abnormalities, including a prominent downbeat nystagmus, that shows partial responsiveness to 4-Aminopyridine (4-AP) [8]. Many patients initially present with ataxic episodes, often leading to the assumption of an autoimmune cause or episodic ataxia.

In contrast to SCA27B, patients with SCA27A initially display predominant tremor, primarily in the upper half of the body, followed by late-onset ataxia and late-onset cognitive decline. These phenotypic core signs are accompanied by interfamilial variability of additional features, including dysarthria, oculomotor impairment with saccadic gaze or, in some cases, nystagmus, dystonia, and sensory neuropathy. Moreover, SCA27A has been associated with psychiatric disorders such as ADHD, autism, and psychosis [5, 7, 9, 12, 13]—a link that is supported by studies in *FGF14*-knockout mice [14].

To date, symptomatic treatment of SCA27A includes beta-blockers and antiepileptic drugs to reduce the tremor. An alternative therapy for tremor and balance is 4-AP, and, anecdotally, deep-brain stimulation (DBS), which has been recently reported in two patients [12].

Notably, fewer than 40 individuals from only a few families with SCA27A have been identified to date. While existing reviews have addressed genetic, clinical, or treatment aspects, none provide a complete overview of all aspects. To highlight the distinct clinical features of SCA27A across genotypes and facilitate clinical comparison with SCA27B, we conducted a comprehensive review of all published cases of SCA27A and report an additional family with SCA27A (Table 1).

**Table 1:** Patient demographics, genetics, and clinical information for all reports to date

Reference		Möller et al. [2026]			Coebergh [2014] [6]	Planes et al. [2015] [15]	Amado et al. [2017] [16]
Patient		Index Patient	Patient 2	Patient 3	Patient	Patient	Twin Sisters
Ethnicity		German	German	German	Netherlands	French	Spanish
Sex		**Female**	**Female**	**Male**	**Male**	**Female**	**Female**
Genotype		*FGF14* (NM_004115.4): c.1_813del (het), *ITGBL1* (NM_019074.4): c.221_1436del (het), *MIR2681* (NR_037498.1): n.1_*del (het) **[chr13:101,520,872–102,020,371]**			***FGF14* (NM_004115.4): c.?_??del (het)** ***ITGBL1* (NM_019074.4): c.??_??del (het) g.101,858,000_102,060,000del (GRCh38, chr13q33.1)**	***FGF14* (ENST00000376131): c.(?_ex2)del (het), g.101,858,000_102,060,000del (GRCh38, chr13q33.1)**	***FGF14* (NM_004115.4): c.?_??del (het)** **g.101,860,000_102,284,000del (GRCh38, chr13q33.1)**
Phenotype							
First symptom		**Hand tremor**	**Hand tremor**	**Hand tremor**	**Poor axial balance**	**Axial ataxia, action tremor of the upper limbs**	**n.r.**
Tremor	AAO [year]	**Childhood**	**4**	**12**	**n.r.**	**Childhood**	**Childhood**
	Intention tremor	**Yes**	**Yes**	**Yes**	**Yes**	**Yes**	**Yes**
	Postural tremor	**Yes**	**Yes**	**No**	**Yes**	**Yes**	**Yes**
	Rest tremor	**Yes**	**Yes**	**No**	**n.r.**	**n.r.**	**Yes**
Ataxia	AAO [year]	**40**	**40**	**20**	**2**	**n.r.**	**Childhood**
	Limb	**Yes**	**Yes**	**Yes**	**Yes**	**Yes**	**Yes**
	Gait	**Yes**	**Yes**	**Yes**	**“Impaired walking”**	**Yes**	**Yes**
	Dysarthria	**Yes**	**Yes**	**Yes**	**Yes**	**n.r.**	**Yes**
	Cerebellar oculomotor signs	**Saccadic gaze**	**Saccadic gaze**	**Saccadic gaze**	**Dysmetric saccades**	**Slow saccades**	**n.r.**
Exacerbation of symptoms		**Deterioration with fever, emotional stress, physical exercise**	**deterioration with emotional stress and physical exercise**	**No**	**Deterioration with fever**	**n.r.**	**Deterioration with fever**
Nystagmus		**Downbeat nystagmus at rest**	**Gaze-evoked nystagmus, upbeat nystagmus**	**Gaze-evoked nystagmus**	**Gaze-evoked nystagmus**	**n.r.**	**Yes**
Dyskinesia		**No**	**No**	**No**	**No**	**n.r.**	**No**
Dystonia		**Torticollis to the left (focal)**	**No**	**Laterocollis to the left (focal)**	**n.r.**	**n.r.**	**Yes**
Reflexes		**Absent ankle reflexes**	**Absent ankle reflexes**	**Absent ankle reflexes**	**No**	**Brisk tendon reflexes**	**n.r.**
Sense of vibration lower body		**Reduced**	**Reduced**	**Reduced**	**No**	**n.r.**	**n.r.**
Sensory neuropathy		**Yes**	**Yes**	**Yes**	**n.r.**	**n.r.**	**n.r.**
Cognition	MoCa	**20/30**	**24/30**	**24/30**	**n.r.**	**n.r.**	**n.r.**
	IQ	**n.r.**	**n.r.**	**n.r.**	**n.r.**	**n.r.**	**low IQ**
Development		**Normal, memory issues**	**Normal, memory issues**	**Normal, memory issues**	**n.r.**	**Developmental delay, microcephaly, convergent strabismus, upslanted palpebral fissure, moderate intellectual disability**	**Developmental delay, executive functions disturbance, memory, learning difficulties**
Neuropsychiatric symptoms		**No**	**Depression**	**Depression**	**n.r.**	**n.r.**	**n.r.**
MRI		**Not performed**	**Normal**	**Post-stroke lesion**	**n.r.**	**Cerebellar atrophy, thin brain stem, T2 and FLAIR hyperintense white matter lesions**	**Normal**
Treatment [effective]		**No**	**No**	**No**	**n.r.**	**n.r.**	**n.r.**
Treatment [not effective]		**4-AP, antiepileptic drugs**	**4-AP, propranolol, antiepileptic drugs**	**No**	**n.r.**	**n.r.**	**n.r.**

Reference		Paucar et al. [2020] [7]						Ceroni et al. [2023] [5]		Hoshina et al. [2003] [17]		Conci et al. [2025] [13]
Patient		Patient (I:1)	Patient II:2	Patient II:5	Patient III:1	Patient III:2	Index Patient IV:1	Family 1 (II.3)	Family 1 (I.1)	Case 1	Case 2	Patient 2
Ethnicity		Swedish						British		n.r.		n.r.
Sex		**Female**	**Male**	**Male**	**Female**	**Female**	**Female**	**Male**	**Male**	**Male**	**Female**	**Male**
Genotype		***FGF14* (NM_004115.4) and ITGBL1 (NM_019074.4): c.?_??del (het)** **g.101,850,000_102,450,000del (GRCh38, chr13q33.1)**						*FGF14* (NM_004115.4): c.-?_*_?del (het) *ITGBL1* (NM_019074.4): c.-?_*_?del (het) g.101,899,000_102,060,000del (GRCh38, chr13q33.1)		**FGF14 (NM_004115.4): c.?*?del (het), g.101,776,915_102,012,410del* (GRCh38, chr13q33.1)**		**FGF14 (NM_004115.4): c.?_??del (het) (chr13q33.1(101887591_101944025)x1)), chr4q22.3 (4q22.3(94693127_95148910)x1 pat 4q22.3PAT8Q24.13**
Phenotype												
First symptom		**Tremor**	**Gait disorder**	**Gait disorder**	**Gait disorder and tremor**	**Dystonia, tremor**	**Tremor**	**Isolated nystagmus**	**Tremor**	**Difficulties in writing**	**Tremor and word-finding difficulties**	**Tremor**
Tremor	AAO [year]	**23**		**n.r.**	**25**	**Neonatal**	**Neonatal**	**Childhood**	**Childhood**	**71**	**46**	
	Intention tremor	**n.r.**	**Tremor, not specified**					**Yes**	**Yes**	**“Shaking legs”**	**Yes**	**29**
	Postural tremor	**Yes**						**n.r.**	**n.r.**		**Yes**	
	Rest tremor	**n.r.**						**n.r.**	**n.r.**		**No**	
Ataxia	AAO [year]	**n.r.**	**30**	**18**	**25**	**Neonatal**	**Neonatal**	**n.r.**	**n.r.**	**67**	**49**	**24**
	Limb	**Yes**	**Yes**	**Yes**	**Yes**	**Yes**	**Yes**	**Yes**	**Yes**	**Yes**	**Yes**	**Yes**
	Gait	**Yes**	**Yes**	**Yes**	**Yes**	**Yes**	**Yes**	**Yes**	**Yes**	**Gait instability**	**Yes**	**Yes**
	Dysarthria	**n.r.**	**n.r.**	**n.r.**	**Yes**	**n.r.**	**n.r.**	**No**	**Yes**	**Yes**	**Yes**	**Yes**
	Cerebellar oculomotor signs	**n.r.**	**n.r.**	**n.r.**	**n.r.**	**n.r.**	**n.r.**	**Vertical saccades**	**Asymmetric horizontal smooth pursuit**	**No**	**Hypermetric saccades**	**n.r.**
Exacerbation of symptoms		**n.r.**	**n.r.**	**n.r.**	**n.r.**	**Deterioration with fever**		**n.r.**	**n.r.**	**Episodic, but no deterioration by stressors**	**Episodic body shaking**	**Deterioration with episodes of trigeminal neuralgia**
Nystagmus		**Yes**	**Yes**	**Yes**	**Yes**	**Yes**	**Yes**	**Horizontal gaze-evoked nystagmus, upbeat nystagmus, rebound nystagmus**	**Yes**	**Downbeat nystagmus**	**Downbeat nystagmus**	**Horizontal and vertical gaze-evoked nystagmus**
Dyskinesia		**n.r.**	**n.r.**	**n.r.**	**n.r.**	**n.r.**	**n.r.**	**n.r.**	**n.r.**	**No**	**No**	**Ballistic movements**
Dystonia		**n.r.**	**n.r.**	**n.r.**	**n.r.**	**Cervical dystonia (focal)**	**n.r.**	**n.r.**	**n.r.**	**n.r.**	**n.r.**	**n.r.**
Reflexes		**Normal**	**Normal**	**Normal**	**Hyporeflexia**	**Normal**	**Normal**	**n.r.**	**n.r.**	**Normal**	**Normal**	**Brisk deep tendon reflexes**
Sense of vibration lower body		**Normal**	**Normal**	**Normal**	**n.r.**	**Normal**	**n.r.**	**n.r.**	**n.r.**	**Normal**	**Normal**	**n.r.**
Sensory neuropathy		**Normal**	**Normal**	**Normal**	**Yes**	**Normal**	**n.r.**	**n.r.**	**n.r.**	**Normal**	**n.r.**	**n.r**
Cognition	MoCa	**n.a.**	**22/30**	**18/30**	**19/30**	**26/30**	**n.r.**	**n.r.**	**n.r.**	**n.r.**	**n.r.**	**n.r**
	IQ	**n.r.**	**n.r.**	**92**	**65**	**71**	**72-82**	**n.r.**	**n.r.**	**n.r.**	**n.r.**	**n.r.**
Development		**Special schools, extra support**	**Special schools, extra support**	**Illiteracy, special schools, extra support**	**Intellectual disability, special schools, extra support**	**Motor and language delay, special schools, extra support**	**Moderate intellectual disability, special schools, extra support**	**Developmental delay**	**n.r.**	**n.r.**	**Word-finding difficulties, cognitive decline**	**Normal development, learning disability**
Neuropsychiatric symptoms		**n.r.**	**Normal**	**Normal**	**BPD, Psychotic syndrome, Depression**	**ADHD, Dyslexia**	**Anger outbursts, ADHD**	**Mood disorder, Aggressiveness**	**Mood disorder**	**n.r.**	**n.r.**	**ADHD, depression, anxiety and aggressive outburst**
MRI		**Mild cortical atrophy**	**Moderate cortical and cerebellar atrophy, mild central atrophy**	**Moderate cortical and mild cerebellar atrophy**	**Moderate cortical and mild central atrophy, subtle atrophy vermis and cerebellum**	**Mild cortical atrophy, mild-moderate atrophy of the vermis, cerebellum, spine**	**Normal**	**Normal**	**n.r.**	**Age-appropriate cerebellar volume loss**	**Normal**	**Normal**
Treatment [effective]		**n.r.**	**n.r.**	**n.r.**	**Neuroleptics**	**n.r.**	**n.r.**	**n.r.**	**n.r.**	**Acetazolamide**	**Acetazolamide**	
Treatment [not effective]		**n.r.**	**n.r.**	**n.r.**	**n.r.**	**Methylphenidate, dexamphetamine**	**n.r.**	**n.r.**	**n.r.**	**n.r.**		**Gabapentin, Carbamazepine, topiramate**

Reference		Van Swieten et al. [2003] [4]	Dalski et al. [2004] [18]	Brusse et al. [2005] [4]		Misceo et al. [2009] [23]	Shimojima et al.[2012]	Tucker et al. [2013] [19]	Choquet et al. [2015] [11]		
Patient		Patient (III:9)	Patient	Patient 1 (III16)	Patient 2 (II.9)	Proband	Patient	Proband	Patient 1 (III.1)	Patient 2 (III.2)	Patient 2 (II.1)
Ethnicity		Dutch	German	Dutch		Norwegian	Japanese	American	French–Canadian		
Sex		Female	Male	Male	Male	Female	Male	Male	Male	Male	Female
Genotype		*FGF14* (NM_004115.4): c.434T>C (p.Phe145Ser) (het) (chr13q33.1))	*FGF14* (NM_004115.4): c.487delA (p.Ser163Alafs*39) (het)	*FGF14* (NM_004115.4): c.434T>C (p.Phe145Ser) (het)(chr13q33.1))		*FGF14* chr13:101,579,849-101,742,909 and chr5:138,866,024-138,903,543 (het), translocation; 46, XX t(5;13)(q31.2;q33.1)	*FGF14* chr13:101,742,909–101,752,504 which disrupted FGF14 46,XY,t(13;21)(q32;q22.3)	De novo, *FGF14* (NM_004115.4): c.1_?del (het), arr[hg18] 13q33.1 (101,171,175–101,268,228)×1	*FGF14* (NM_004115.4): c.211_212insA (p.Ile71Asnfs*27) (het)		
Phenotype											
First symptom		Hand tremor	n.r.	Hand tremor	Postural hand tremor	n.r.	Muscle tonus	Tremor	Unsteady gait, dysarthria, vertical oscillopsia	Unsteady gait, dysarthria, vertical oscillopsia	Incoordination
Tremor	AAO [year]	Childhood	13	Childhood	20	<1	n.r.	<1	29	n.r.	n.r.
	Intention tremor	Hand tremor	Hand tremor	Yes	n.r.	Yes	n.r	Tremor	Yes	Yes	Normal
	Postural tremor			Yes	yes	Yes	n.r		Yes	Yes	Normal
	Rest tremor			Yes	n.r.	No	n.r		No	n.r.	Normal
Ataxia	AAO [year]	28	12	30	27	<1	n.r.	4.5	26	n.r.	n.r.
	Limb	n.r.	Yes	Yes	Yes	n.r.	n.r.	n.r	Yes	n.r.	Normal
	Gait	Yes	Yes	Yes	Yes	Yes	n.r.	Yes	Yes	Yes	Yes
	Dysarthria	Yes	n.r.		Yes	Yes	n.r.	Yes	Yes	Yes	Normal
	Cerebellar oculomotor signs	Dysmetric saccades	n.r.	Slow saccades	n.r.	n.r.	n.r.	n.r	n.r.	n.r.	Normal
Exacerbation of symptoms		Deterioration with emotional stress and physical exercise	n.r.	Deterioration with physical or emotional stress	n.r.	Yes	n.r.	n.r	Deterioration with fever and exercise	Deterioration with fever and exercise	n.r.
Nystagmus		Gaze-evoked nystagmus	Gaze-evoked nystagmus	Gaze-evoked nystagmus	n.r.	No	n.r	n.r	Horizontal nystagmus	Upbeat and downbeat nystagmus	Horizontal nystagmus
Dyskinesia		No	n.r.	n.r.	Orofacial dyskinesia	Dyskinetic jerky neck and arm movements	Episodic involuntary movements	No	n.r.	n.r.	n.r.
Dystonia		n.r.	n.r.	n.r.	n.r.	n.r.	n.r	No	Right arm (segmental)	n.r.	n.r.
Reflexes		n/a	n.r.	Brisk tendon reflex, normal plantar reflex	n.r.	Increased tendon reflexes, normal plantar reflexes	n.r	Normal	n.r.	n.r.	n.r.
Sense of vibration lower body		n/a	n.r.	Normal	Reduced	n.r.	n.r	Normal	n.r.	n.r.	n.r.
Sensory neuropathy		n.r.	Yes	Normal	n.r.	n.r.	n.r	Normal	Normal	n.r.	n.r.
Cognition	MoCa	n.r.	n.r.	n.r.	n.r.	n.r.	n.r	n.r	n.r.	n.r.	n.r.
	IQ	n.r.	70	79	10-25th percentile	n.r.	n.r	77	n.r.	n.r.	n.r.
Development		n.r.	Pes cavus inborn strabismus, mild mental retardation	n.r.	n.r.	Short neck, clinodactyly, high-arched feet, mental retardation	Mild mental retardation	Mildly dysmorphic, with mild acrocephaly, motor skill delay, speech delay	n.r.	n.r.	n.r.
Neuropsychiatric symptoms		No	n.r.	n.r.	n.r.	n.r.	n.r.	n.r.	n.r.	n.r.	n.r.
MRI		Normal	Normal	Normal	Moderate cerebellar atrophy	Normal	Normal	n.r.	Normal	n.r.	n.r.
Treatment [effective]		n.r.	n.r.	n.r.	n.r.	n.r.	n.r.	n.r.	n.r.	n.r.	n.r.
Treatment [not effective]		n.r.	n.r.	Alcohol, propranolol, dopaminergic medication		n.r.	Valproic acid, phenobarbital	n.r.	Acetazolamide	n.r.	Acetazolamide

Reference		Groth et al [2018] [9]	Miura et al. [2019] [21]	Schesny [2019] [22]	Piarroux et al. [2019] [20]		Keller et al. [2025] [12]			Conci et al. [2025] [13]
Patient		Patient	Patient	Patient	Index Patient (Family A, IV-10)	Index Patient (Family B, II.2)	Patient 1 (III2)	Patient 1 (III3)	Patient 3 (III1)	Patient 1
Ethnicity		American	Japanese	Switzerland	French	French	American			n.r
Sex		Male	Male		Male	Female	Female	Female	Female	Male
Genotype		*FGF14* (NM_004115.4): c.326T>C (p.Phe109Ser)(het)	*FGF14* (NM_004115.4): c.529A>T (p.Lys177) (het)	*FGF14* (NM_175929.2): c.208+1G>A (het)	*FGF14* (NM_004115.3): c.439G>T (p.Glu147) (het)	*FGF14* (NM_004115.3): c.486_487del (p.Tyr162) (het)	*FGF14* (NM_004115.4/ENST00000376131): c.356_358del (p.Val119del)(het)			FGF14 (NM_004115.4): c.?_??del (het) (chr13q33.1(101887591_101944025)x1)), chr4q22.3 (4q22.3(94693127_95148910)x1 pat 4q22.3PAT8Q24.13
Phenotype										
First symptom		Tremor right hand	Gait disturbance	Episodic vertigo	Nystagmus, ataxia	Nystagmus	Action hand tremor	Hand tremor	Hand tremor	No
Tremor	AAO [year]	20	n.r.	n.r.	n.r.	1	12	3	12	14 month
	Intention tremor	Yes	Yes	n.r.	n.r.	Yes	Yes	Yes	Yes	n.r.
	Postural tremor	Yes	Yes	n.r.	n.r.	Yes	Yes	Yes	Yes	n.r.
	Rest tremor	Yes	Yes	n.r.	n.r.	No	Yes	No	No	n.r.
Ataxia	AAO [year]	8	47	20s	1	1	Mid 40s	42	30s	n.r.
	Limb	Yes	Yes	Yes	Yes	Yes	Yes	Yes	n.r.	n.r.
	Gait	Yes	Yes	Yes	Yes	Yes	Yes	Yes	yes	No, poor sitting balance
	Dysarthria	Yes	yes	n.n	n.r.	n.r.	Yes	Yes	yes	n.r.
	Cerebellar oculomotor signs	No	Saccadic dysmetria, intrusions	Saccadic gaze	n.r.	n.r.	Slow saccades	Saccadic pursuit	n.r.	n.r. [abnormal eye movements]
Exacerbation of symptoms		Yes	n.r.	Deterioration with stress, physical activity, caffeine	Deterioration with fever	Deterioration with fever	n.r.	Yes	Yes	Episodic deterioration, often triggered by stress
Nystagmus		Gaze-evoked nystagmus, upbeat nystagmus	n.r.	Gaze-evoked nystagmus, rebound nystagmus	Multidirectional	Vertical nystagmus	No	Gazed-evoked downbeat nystagmus	Horizontal nystagmus, upbeat nystagmus	n.r. [abnormal eye movements]
Dyskinesia		No	n.r.	n.r.	n.r.	n.r.	n.r.	n.r.	n.r.	Bilateral trunk and upper limb twitching
Dystonia		No	n.r.	n.r.	n.r.	n.r.	n.r.	Right hand dystonia (focal)	Dystonia in the upper extremities (segmental)	n.r.
Reflexes		Normal	Reduced ankle reflexes	Normal	n.r.	n.r.	n.r.	n.r.	n.r.	Normal
Sense of vibration lower body		Normal	Reduced in lower limbs	n.r.	n.r.	n.r.	n.r.	n.r.	n.r.	Normal
Sensory neuropathy		n.r.	n.r.	n.r.	n.r.	n.r.	n.r.	n.r.	n.r.	Normal
Cognition	MoCa	n.r.	n.r.	n.r.	Normal	n.r.	n.r.	n.r.	n.r.	n.r.
	IQ	n.r.	n.r.	n.r.	Normal	n.r.	n.r.	n.r.	n.r.	n.r.
Development		Secondary school	Junior high school	Motor developmental delay	Normal	Global delay, learning difficulties	n.r.	n.r.	Attention and memory issues	Plagiocephaly and right pseudoesotropia, normal developmental milestones
Neuropsychiatric symptoms		PTSD, depression	n.r.	Normal	n.r.	n.r.	n.r.	n.r.	Fatigue, lightheadedness	Emotional outbursts
MRI		n.r.	Cerebellar atrophy	Cerebellar atrophy	n.r.	Normal	Mild vermian atrophy	n.r.	n.r.	Normal brain and spine
Treatment [effective]		Amantadine, levodopa/carbidopa	n.r.	Acetazolamide, chlorzoxazone	n.r.	n.r.	4-AP, STN-DBS	STN-DBS, Botulinum toxin	4-AP	No medication
Treatment [not effective]		Ropinirole, propranolol	n.r.	Chlorzoxazone	n.r.	n.r.	n.r.	n.r.	n.r.	No medication

The bold highlighted columns represent a deletion spanning both the *FGF14* and *ITGBL1* genes. The genotype was specified as far as known at the cDNA and pDNA level. Unknown deletion sites were marked with a “?”. Sensory neuropathy, if confirmed by nerve conduction studies or explicitly reported in the publication.

*N.R* Not reported, *N.A.* Not accessed, *AAO* Age at onset, * leading to a stop codon, *?* unknown exact cDNA level, *del* deletion, *het* heterozygous, *FGF14* Fibroblast growth factor 14, *ITGBL1* Integrin subunit beta-like 1, *MIR2681* MicroRNA 2681, *IQ* Intelligence quotient, *BPD* Borderline personality disorder, *ADHD* Attention-deficit-hyperactivity disorder, *PTSD* Post-traumatic stress disorder, *4-AP* 4-Aminopyridine, *STN*-*DBS* Subthalamic nucleus deep-brain stimulation, *FLAIR* Fluid-attenuated inversion recovery

Methods: A literature search and data extraction for publications on individuals with SCA27A were conducted using standard search terms (“spinocerebellar ataxia 27A,” “SCA27A”, and “spinocerebellar ataxia type 27A”) in the NCBI PubMed database (https://pubmed.ncbi.nlm.nih.gov/). Titles, abstracts, and full texts of peer-reviewed original articles written in English and published up to November 8th, 2025, were screened for eligibility. Only studies reporting at least one individual with a genetically confirmed pathogenic *FGF14* variant consistent with SCA27A were included. Preprint articles were excluded from the analysis. In addition, the OMIM database (www.omim.org) was consulted to identify further publications related to SCA27A (OMIM #193003). Any references not captured during the initial search were retrieved through backward searches in PubMed and, when relevant, included. The literature summary is structured chronologically by year of publication, with the exception that individuals carrying an *FGF14* deletion larger than 200 kb—including cases reporting a combined deletion of *FGF14* and parts of *IGTBL1*—were grouped together. Table 1 displays only index patients and family members who underwent comprehensive clinical and genetic evaluation. Additional individuals mentioned only in overview tables and without detailed characterization were excluded (Table 1).

**Case reports:** We report three out of four affected siblings of a four-generation German family. The family also includes a deceased affected father and grandfather, who could not be genetically tested. One of our index patients’ sons (IV 7) was reported to have tremor and gait difficulties, but was unavailable for genetic testing and clinical examination. Patient 2’s son (IV 4) was reported to suffer from a confirmed SCA27A, although his genetic testing results and blood were not available to us (Fig. 1a).

**Fig. 1:**
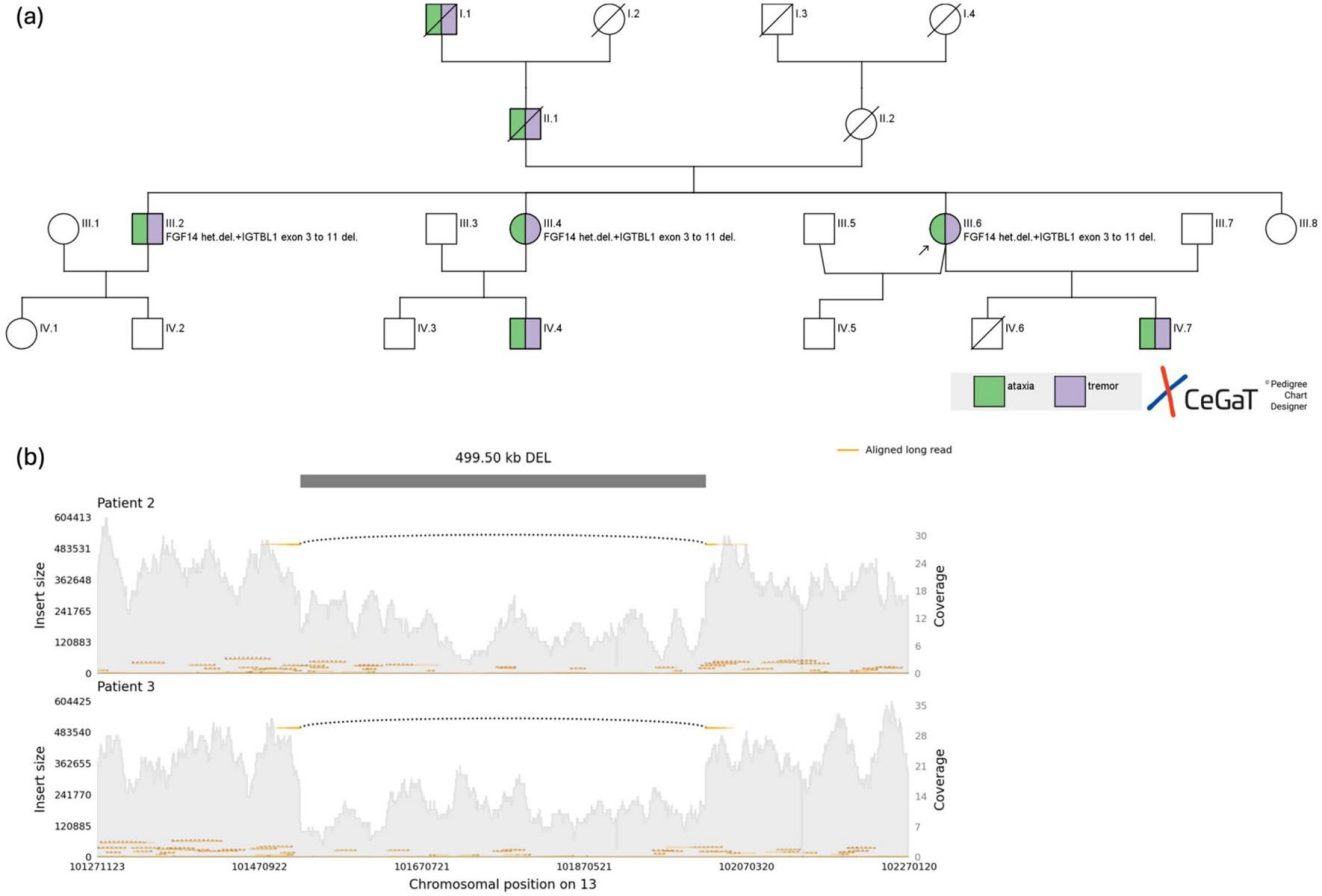
**a** Pedigree of a three-generation German family with SCA27A. Green-shaded symbols indicate individuals with ataxia; purple-shaded symbols indicate individuals with tremor. Patients III.2, III.4, and III.6 have genetically confirmed diagnoses, as indicated. Patient I.1 and II.2 seemed to be affected but were not genetically tested. Patient IV.4 is reported as affected and has undergone genetic testing; patient IV.6 is reported as affected, but neither has been seen by our team. The arrow denotes the index patient. The visualization tool CeGat was used. **b** Long-read sequencing results. Yellow lines represent aligned long reads; black dots indicate the detected deletions (split read). Both deletions are located on chromosome 13. The exact coordinates of the deletion in Patient 2 are chr13:101,520,872–102,020,371. The visualization tool Samplot was used [25]

Our 55-year-old female index patient presented with upper limb and head tremor in childhood, which progressively worsened over time and was accompanied by focal dystonic posturing. The tremor was exacerbated by stress, fever, and cognitive load. In her 40s, gait disturbances and cognitive difficulties began to become apparent. Neurological examination indicated mild cognitive impairment, with a score of 20/30 on the Montreal Cognitive Assessment (MoCA), taking into account 10 years of education. Developmental milestones had been reached without delay; however, upon questioning, the patient reported experiencing cognitive difficulties, such as word-finding problems, over several years, with slight progression. Oculomotor assessment demonstrated saccadic eye pursuit and a subtle downbeat nystagmus exacerbated on lateral gaze. The motor examination revealed cerebellar signs, including dysmetria with intention tremor, pronounced bradydysdiadochokinesia, a broad-based ataxic gait and stance (Video 1). Mild distal sensory neuropathy was also present.

Her 65-year-old sister reported tremor onset at age 4 years, progressively worsening and later involving the head and trunk. From the age of 40 years, she developed gait disturbance with frequent falls and, over the past 2 years, progressive cognitive decline affecting both short- and long-term memory. Their 58-year-old brother developed tremor at the age of 12 years, initially presenting as fine motor difficulties, followed by slowly progressive gait instability from his 20s and mild cognitive complaints. Patients 2 and 3 had mild cognitive impairment (MoCA 24/30). Oculomotor findings included a subtle downbeat nystagmus in Patient 3, evoked on horizontal gaze, as well as impaired VOR in Patients 2 and 3. Both exhibited an unsteady ataxic gait with additional cerebellar signs. Tremor was more severe and widespread in Patient 2, whereas Patient 3 exhibited a milder upper limb tremor, accompanied by a focal dystonic laterocollis (Video 2 and 3). Patient 3 also exhibited mild hemihypesthesia following a stroke approximately 1 year prior to examination, without associated motor deficits. Additionally, both had absent Achilles tendon reflexes and a remarkably reduced sense of vibration.

All siblings underwent genetic testing in different settings: Array-CGH of Patient 2 and our index patient was performed in a clinical setting and revealed a heterozygous 500 kb deletion of the *FGF14* gene (exon 1 to 5) and a part of the neighboring *ITGBL1* gene (exon 3 to 11) and MicroRNA gene (*MIR2681*) of the Index patient. Additionally, we performed long-read sequencing on a research basis that confirmed a heterozygous in-frame 500 kb deletion affecting *FGF14*, located on chromosome 13q33 in both Patients 2 and 3 (Fig. 1b). The position of the deletion spans the gene from 101,520,872 to 102,020,371 on chromosome 13 (GRCh38) in Patient 2.

**Literature review and discussion:** In this study, we provide a comprehensive synthesis of all published SCA27A reports to date and add a multigenerational German family with a heterozygous deletion involving *FGF14* and parts of *ITGBL1* and *MIR2681* (Table 1).

Across the literature and our family, a consistent core phenotype emerges, characterized by early-onset tremor, followed by slowly progressive cerebellar ataxia and mild cognitive impairment, with additional frequent features such as nystagmus, dystonia, and sensory neuropathy [5–7, 13, 15–17]. These findings refine the clinical spectrum of SCA27A and help to delineate it more clearly from SCA27B and other *FGF14*-related disorders.

Despite marked mutational heterogeneity—including microdeletions [12, 13, 18–20], missense [4, 9, 10] and nonsense variants [20, 21], frameshift [11] and splice-site mutations [22], and structural rearrangements [23, 24]—most reported individuals with SCA27A share a similar constellation of early tremor, cerebellar dysfunction, and variable cognitive involvement. Our family fits well into this pattern and adds further evidence that deletions extending beyond *FGF14* into *ITGBL1* and *MIR2681* do not produce a clearly distinguishable clinical phenotype, suggesting that haploinsufficiency of *FGF14* is the main disease driver. At the same time, intra-familial variability in age at onset, progression rate, and severity of tremor, ataxia, and cognitive dysfunction in our kindred illustrates the broad expressivity that has been noted across prior reports [4–7, 9–13, 15–24] (Table 1).

Our comparison of SCA27A and SCA27B underscores that these entities, while sharing a common genetic locus, occupy distinct positions within a broader *FGF14*-related spectrum. SCA27A is typically defined by early-onset tremor, earlier and more steadily progressive cerebellar ataxia, and a higher frequency of cognitive impairment and additional movement disorders, including dystonia. In contrast, SCA27B, caused by intronic GAA repeat expansions, usually manifests as late-onset, often episodic cerebellar ataxia with prominent downbeat nystagmus and without early tremor or overt cognitive decline, although nystagmus and gait disturbance represent shared features across both conditions and may respond to 4-AP [8, 12].

Clinically, recognition of early tremor in combination with slowly progressive ataxia, subtle but characteristic oculomotor abnormalities, and mild cognitive decline should prompt consideration of SCA27A, especially in autosomal-dominant families with variable age at onset and additional movement disorders. This has direct diagnostic implications, as such constellations may easily be misattributed to essential tremor, Parkinson’s disease, hereditary neuropathies, autoimmune ataxias, or other genetic ataxias, and therefore support early, targeted testing of *FGF14*, including structural variants and, where appropriate, whole-genome sequencing. From a therapeutic perspective, our patients highlight that 4-AP may not be universally effective, as none showed a clinical response, particularly given that downbeat nystagmus is only mild. Beyond this, DBS represents an emerging therapeutic option for severely disabling tremor in SCA27A and warrants systematic evaluation in larger cohorts [12].

This work is limited by the reliance on published case reports and small series with heterogeneous clinical assessments, incomplete neuropsychological data, and variable reporting of sensory and neuropsychiatric features. Our own contribution is likewise constrained by the modest number of affected family members available for detailed evaluation and the absence of systematic longitudinal cognitive and imaging studies. Future research should focus on prospective, deeply phenotyped cohorts of *FGF14*-related disorders to clarify genotype–phenotype correlations, better quantify cognitive and neuropsychiatric involvement, and systematically evaluate targeted therapies, including 4-aminopyridine and deep-brain stimulation.

These findings underscore the value of precise phenotyping—a distinctive contribution of this study.

## Supplementary Information

Below is the link to the electronic supplementary material.

Supplementary file1 (MP4 249793 kb)

Supplementary file2 (MP4 283809 kb)

Supplementary file3 (MP4 249590 kb)

Supplementary file4 (PDF 154 kb)

## Data Availability

The datasets analyzed during the current study are stored on a secure internal server in accordance with approved ethics protocols. Associated biomaterials are stored in our institutional biobank at the Institute of Neurogenetics, University of Lübeck, Germany. The biobank is integrated as an external interface within the quality management system of the Interdisciplinary Center for Biobanking Lübeck (ICB-L) and is covered by ethics committee approval. Data and materials are available upon reasonable request and in accordance with ethical regulations.
