# Supplementary material for: Phenotypic spectrum of FGF14-related late-onset ataxia: predominant tremor and cognitive decline as key features of SCA27A
Source: J Neurol. 2026 May 13;273(6):309. doi: 10.1007/s00415-026-13813-1 (PMC13171637; doi:10.1007/s00415-026-13813-1)
Supplement: Supplementary file 4 — Supplementary file4 (PDF 154 kb) [file 415_2026_13813_MOESM4_ESM.pdf]

Phenotypic Spectrum of *FGF14*-Related Late-Onset Ataxia: Predominant Tremor and Cognitive Decline as Key Features of SCA27A

Meret Möller[1], André Fienemann[2], Joanne Trinh[2], Christoph Much[2], Björn H. Falkenburger[3, 4], Iñaki Schniewind[3,4], Yorck Hellenbroich[5], Norbert Brüggemann[1], Christine Klein[1,2], Alexander Balck[1,2]

1 Section for Movement Disorders, Department of Neurology, University of Lübeck, University Hospital Schleswig-Holstein, Campus Lübeck, Lübeck, Germany
2 Institute of Neurogenetics, University of Lübeck, Lübeck, Germany
3 Department of Neurology, University Hospital Carl Gustav Carus, TUD Dresden University of Technology, Dresden, Germany
4 German Center for Neurodegenerative Diseases (DZNE), Dresden, Germany
5 Department of Human Genetics, University Hospital Schleswig-Holstein, Lübeck, Germany.

Corresponding author: alexander.balck@uni-luebeck.de

Supplement

Video 1

Index Patient. Neurological examination showing tremor, gait ataxia, dysmetria, dysdiadochokinesia, and eye movements,

Video 2

Patient 2. Neurological examination showing tremor, gait ataxia, dysmetria, dysdiadochokinesia, and eye movements.

Video 3

Patient 3. Neurological examination showing dysarthria, tremor, gait ataxia, and nystagmus. Ptosis is congenital.
